# Biomarker Value of miR-221 and miR-222 as Potential Substrates in the Differential Diagnosis of Papillary Thyroid Cancer Based on Data Synthesis and Bioinformatics Approach

**DOI:** 10.3389/fendo.2021.794490

**Published:** 2022-02-07

**Authors:** Shang Cai, Jiayan Ma, Yong Wang, Yuxing Cai, Liwei Xie, Xiangying Chen, Yingying Yang, Qiliang Peng

**Affiliations:** ^1^ Department of Radiotherapy & Oncology, The Second Affiliated Hospital of Soochow University, Suzhou, China; ^2^ Institute of Radiotherapy & Oncology, Soochow University, Suzhou, China; ^3^ Department of Experimental Center, The Second Affiliated Hospital of Soochow University, Suzhou, China

**Keywords:** papillary thyroid cancer, miR-221/222 cluster, diagnostic value, biomarker, bioinfomatics

## Abstract

**Background:**

MicroRNA (miRNA) has been reported to play a critical regulatory role in papillary thyroid carcinomas (PTC). However, the role of miR-221/222 in PTC remains unclear. Here, we performed this study to explore the diagnostic potentials and mechanisms of miR-221/222 in PTC.

**Methods:**

First, we systematically analyzed the diagnostic value of miR-221/222 in the diagnosis PTC by pooling the published studies. Afterwards, we performed comprehensive bioinformatics analysis including gene ontology analysis, pathway enrichment analysis and protein-protein interaction analysis to explore the potential mechanisms of miR-221/222 involved in PTC.

**Results:**

The overall sensitivity and specificity of miR-221/222 for PTC were 0.75 (95% CI: 0.70–0.80) and 0.80 (95% CI: 0.76–0.84) respectively with the AUC of 0.85 (95% CI: 0.81-0.88). The diagnostic performance varied among different subgroups including geographical locations, sample sources and sample sizes. Meanwhile, we found that a combination of miR-221/222 and other miRNAs when used in a diagnostic panel could improve the diagnostic accuracy than individual miR-221/222. Moreover, through the bioinformatics analysis, we confirmed that miR-221/222 targets were highly related to the molecular pathogenesis of PTC. The results revealed that miR-221/222 may exert important functions in PTC through thyroid hormone signaling pathway and some other key pathways by regulating some key genes.

**Conclusion:**

These findings indicated that miR-221/222 have the potential to serve as auxiliary tools for diagnosing PTC. Further prospective clinical trials should be performed to assess the accuracy of these findings in a larger cohort and determine the clinical uses.

## Introduction

Thyroid carcinoma (TC) represents the most frequent carcinoma of the endocrine system with a rapidly rising incidence for the past three decades (1). According to the histological features, TC can be subdivided into four classes, including papillary thyroid carcinoma (PTC), follicular thyroid carcinoma (FTC), poorly differentiated carcinoma (PDC) and anaplastic thyroid carcinoma (ATC) (2). PTC represents the most frequent TC (accounting for approximately 85%) (3). Currently, the diagnostic methods of PTC mainly depend on ultrasound, computed tomography (CT) and fine-needle aspiration cytology (4). However, these procedures are not easy and require professional performance of biopsy sampling and well-prepared histological micropreparations, which can be difficult in some cases. Therefore, it is still in urgently needed to search sensitive and objective biomarkers for assisting in diagnosis of patients with PTC.

MicroRNAs (miRNAs) are a class of endogenous non-coding RNA molecules with a wide range of functions involved in a variety of biological processes, including differentiation, proliferation, apoptosis and carcinogenesis (4). A large number of studies have shown that miRNAs can serve as potential biomarkers for human disease and as targets for disease intervention (5). Besides, miRNAs can be stably measured in various types of sample sources, making them possess promising biomarker characteristics (6). Moreover, recent evidence has revealed that the expression of miRNA is highly associated with the occurrence and development of PTC and can be employed as a molecular biomarker for its diagnosis, treatment and prognosis (7). Therefore, identifying miRNAs that may serve as potential markers of PTC after an initial screening or diagnostic test has a significant clinical value.

Interestingly, miR-221 and miR-222 are two of the typical and most extensively studied examples in functional miRNAs in thyroid carcinoma (8). They are highly homologous as they are encoded on chromosome X (Xp11.3) and share seed sequences (9). Increasing evidence has indicated that miR-221 and miR-222 are frequently deregulated across different cancer types including PTC (10). Moreover, high expression of miR-221 and miR-222 significantly predicted poor OS in cancer prognosis (11). Liang et al. performed a meta-analysis to observe whether a difference in miRNA-221/222 expression exists in thyroid cancer with normal thyroid or BTLs and found that they were up-regulated in PTC (12). Besides, it was revealed from recent new evidence that miR-221 and miR-222 play a key role in the occurrence and development of PTC (13). Zhang et al. indicated that the miR-222 and its critical target genes could be promising biomarkers for the prognosis of thyroid cancer and may act as important regulators of immune infiltration in thyroid cancer (14). In addition, the diagnostic value of miR-221/222 expression in PTC has been extensively evaluated in the past decade. Nevertheless, their diagnostic role and potential mechanisms in PTC patients have not been fully analyzed and investigated. In addition, single studies may be underpowered to achieve a comprehensive and reliable conclusion.

Therefore, to comprehensively understand whether miR-221 and miR-222 could serve as diagnostic biomarkers for PTC, we conducted a systematic meta-analysis to assess the diagnostic efficiency of miR-221/222 in PTC patients from published literatures, combined with a bioinformatics study, and to identify the potential mechanisms of them involved in PTC.

## Methods

### Literature Search Strategy

A computerized search of the PubMed, EMBASE and Web of Science databases was conducted to find relevant, original literature reports on the use of miR-221/222 to diagnose patients with PTC. Our search was based on the following key terms: “microRNA-221 or microRNA-222 or miRNA-221 or miRNA-222 or miR-221 or miR-222” and “thyroid neoplasm or thyroid carcinoma or thyroid cancer or thyroid tumor” and “papillary” and “sensitivity or specificity or true positive rate or false positive rate or diagnosis or diagnostic or diagnose” in titles or abstract (up to Feb. 2021). Simultaneously, manual searching of related citations and reference lists was undertaken to obtain other potential studies.

### Eligibility Criteria

Eligible studies included in the meta-analysis part adhered to the following criteria: (1) miR-221 or miR-222 was used to diagnose PTC; (2) pathological examination was used as reference standard; (3) sufficient information was provided to construct a 2 × 2 table to calculate true positives (TP), true negatives (TN), false positives (FP) and false negatives (FN).

The exclusion criteria were as follows: (1) reviews, letters, comments, or conferences abstracts; (2) studies without sufficient information to calculate the above parameters; (3) studies with duplicated data; (4) full texts unavailable for review.

### Data Extraction and Quality Assessment

Data were extracted independently in standardized data collection forms. The extracted data included the following details: 1) study characteristics: authors, year of publication, country; 2) demographic and clinical characteristics of the patients: ethnicity, mean age, number of participants; 3) miRNA characteristics: sample source, detected miRNAs, detection methods; and 4) the diagnostic performance of miR-221/222 in PTC: sensitivity, specificity, area under the ROC curve (AUC).

The revised quality assessment of diagnostic accuracy studies (QUADAS-2) tool was used to assess the quality of the included studies (15).

Two reviewers (S.C., J.M.) independently read the titles and abstracts of the included articles, judged their eligibility and evaluated the quality of each article. Any disagreements between reviewers were resolved through discussion between the authors or by consultation with the third reviewer (Y.W.).

### Data Synthesis and Analyses

We applied the bivariate mixed-effects regression model to calculate the pooled sensitivity, specificity, positive likelihood ratio (PLR), negative likelihood ratio (NLR), diagnostic odds ratio (DOR) and the corresponding 95% CIs (16). A heterogeneity analysis was performed based on the Cochran Q test and the Higgins I square test (17). If P <0.05 or I^2^ > 50%, a random-effects model was selected. Otherwise, a fixed-effects model was employed. A summary receiver operating characteristic curve (SROC) was drawn based on the literature, and the area under the SROC curve (AUC) was calculated for the overall evaluation of diagnostic performance (18). The threshold effect was checked by using the spearman correlation coefficient. The factors contributed to heterogeneities were analyzed by subgroup analysis and meta-regression analysis (19). Afterwards, the stability of the results was assessed by sensitivity analysis. Potential publication bias was analyzed by funnel plot (20). All analyses were performed using STATA 14.0 software. The statistical significance threshold for all analyses was set at 0.05.

### Target Genes Prediction of miR-221/222

The target gene prediction for miR-221 and miR-222 was performed by using the online miRTarBase target gene prediction database. The miRTarBase program accumulated >13,404 validated miRNA-target interactions, which were collected from 11,021 articles, and calculated an integrative score for any of those predicted target genes (21).

### Functional and Pathway Enrichment Analysis

To characterize the biological function associated with miR-221/222, gene ontology (GO) and Kyoto Encyclopedia of Genes and Genomes (KEGG) pathway analyses were performed, which are two common enrichment analysis methods to determine the high-level functions and uses of cells and organisms from their genomic information (22, 23). GO analysis contains biological processes (BP), cellular component (CC), and molecular function (MF) analyses. In our study, the GO and KEGG pathway analyses were performed using the online software, Database for Annotation, Visualization and Integrated Discovery (DAVID), version 6.8 (https://david.ncifcrf.gov/), which provides a comprehensive set of functional annotation tools to clarify the biological relevance of target genes and pathways (24). The selection criteria for significant items were in accordance with P<0.05 and gene count number ≥2.

### PPI Network Construction and Module Analysis

In order to explore the mutual associations among the target genes of miR-221/222, all the targets of miR-221/222 were imported into the Search Tool for the Retrieval of Interacting Genes (STRING) website for further analysis (25). In our study, we only selected the PPI data validated by text-mining or experiments with the combined score>0.4. Subsequently, Cytoscape software was used to visualize the PPI network and select the key target genes and modules. The plug-in CytoNCA was utilized to calculate the network properties such as betweenness centrality, closeness centrality, and degree centrality to determine the hub genes (26). Significant modules in the PPI network were then identified by molecular complex detection (MCODE) (27). Finally, in order to further analyze the functions of module genes, KEGG enrichment analysis of these genes were performed using the DAVID online website. P-values < 0.05 were considered to be significant.

### Validation of Hub Genes

First, KEGG pathway enrichment analysis was performed to elucidate the potential functions of the identified hub genes. Subsequently, a literature search regarding the associations among the pathways and thyroid cancer or PTC in PubMed was performed. In addition, the analysis of relative expression of the identified hub genes was performed using Gene Expression Profiling Interactive Analysis (GEPIA), which is a valuable and highly cited resource for gene expression analysis based on tumor and normal samples from the TCGA and the GTEx databases (28). In our study, we employed the boxplot to visualize the mRNA expression of the identified hub genes in thyroid tissues and normal tissues.

## Results

### Search Results and Characteristics of Studies

**Figure 1:**
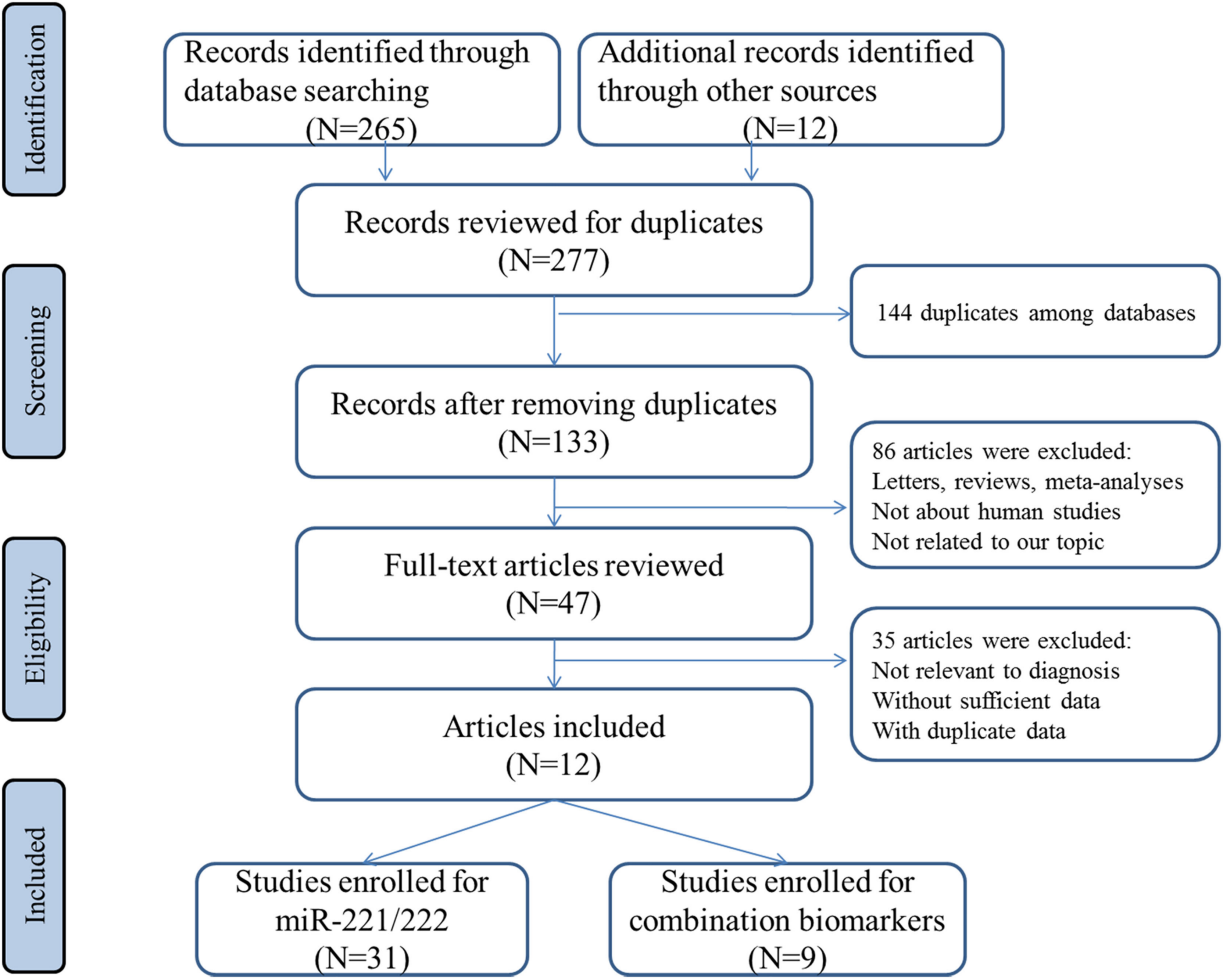
Flowchart of the study selection.

The flowchart for the selection of eligible studies was illustrated in **Figure 1**. By using the described searching strategy, we primarily collected 277 citations. In total, 47 full-text articles were retrieved and examined for relevance. Finally, a total of 12 articles containing 31 studies fulfilled the inclusion criteria and were included (29–40).

**Table 1** provides the main characteristics of the 31 included studies. The thirty-one included studies were published between 2011 and 2020. The majority of the studies were performed in Asia (20/31, 64.5%; 16 in China) and the others were conducted in Europe (11/31, 35.5%). All the 31 studies used the quantitative real-time reverse transcription-PCR (RT-PCR) method to measure the expression of miRNAs, with histopathological examination as the gold standard for PTC diagnosis. Fifteen studies detected the expression of miRNAs in tumor tissues, while sixteen studies measured miRNAs in serum or plasma. The qualities of the 31 studies were methodologically evaluated using QUADAS-2. As a whole, the qualities of included studies are satisfying and eligible.

**Table 1:** The main features of the included studies for miR-221/222 in the diagnosis of PTC.

Author	Year	Country	Ethnicity	Patients			Control		N	miRNA	Source	Methods	AUC	Sensitivity	Specificity
				Np	Age	Stage	Nc	Age							
Mazeh et al. (29)	2011	Israel	Asian	20	45	NA	7	45	27	miR-221	Tissue	qRT-PCR	NA	0.93	0.92
Mazeh et al. (29)	2011	Israel	Asian	20	45	NA	7	45	27	miR-222	Tissue	qRT-PCR	NA	0.88	0.90
Pai et al. (30)	2012	India	Asian	22	NA	NA	11	NA	33	miR-221	Tissue	qRT-PCR	0.862	0.86	0.91
Pai et al. (30)	2012	India	Asian	22	NA	NA	11	NA	33	miR-222	Tissue	qRT-PCR	0.792	0.82	0.91
Yu et al. (31)	2012	China	Asian	106	45	I-IV	95	45	221	miR-222	Serum	qRT-PCR	0.906	0.81	0.89
Yu et al. (31)	2012	China	Asian	106	45	I-IV	44	45	150	miR-222	Serum	qRT-PCR	0.882	0.94	0.70
Sun et al. (32)	2013	China	Asian	52	45	I-IV	52	45	104	miR-221	Tissue	qRT-PCR	0.872	0.83	0.79
Sun et al. (32)	2013	China	Asian	52	45	I-IV	52	45	104	miR-222	Tissue	qRT-PCR	0.868	0.71	0.88
Panebianco et al. (33)	2015	Italy	Caucasian	38	NA	NA	13	NA	51	miR-222	Tissue	qRT-PCR	0.551	0.48	0.69
Rosignolo et al. (34)	2016	Italy	Caucasian	44	46	I-IV	19	46	65	miR-221	Serum	qRT-PCR	0.73	0.40	0.96
Rosignolo et al. (34)	2016	Italy	Caucasian	44	46	I-IV	19	46	65	miR-222	Serum	qRT-PCR	0.587	0.48	0.84
Rosignolo et al. (34)	2016	Italy	Caucasian	44	46	I-IV	20	NA	66	miR-221	Serum	qRT-PCR	0.931	0.88	0.85
Rosignolo et al. (34)	2016	Italy	Caucasian	44	46	I-IV	20	NA	66	miR-222	Serum	qRT-PCR	0.852	0.91	0.70
Titov et al. (35)	2016	Russia	Caucasian	108	53	I-IV	108	53	216	miR-221	Tissue	qRT-PCR	NA	0.81	0.83
Titov et al. (35)	2016	Russia	Caucasian	108	53	I-IV	108	53	216	miR-222	Tissue	qRT-PCR	NA	0.76	0.92
Xu et al. (36)	2016	China	Asian	59	47	NA	15	47	74	miR-221	Tissue	qRT-PCR	0.633	0.71	0.61
Xu et al. (36)	2016	China	Asian	59	47	NA	15	47	74	miR-222	Tissue	qRT-PCR	0.615	0.69	0.59
Zhang et al. (37)	2017	China	Asian	85	45	I-IV	35	45	120	miR-221	Serum	qRT-PCR	0.704	0.86	0.53
Zhang et al. (37)	2017	China	Asian	85	45	I-IV	35	45	120	miR-222	Serum	qRT-PCR	0.840	0.63	0.88
Zhang et al. (37)	2017	China	Asian	85	45	I-IV	40	45	125	miR-221	Serum	qRT-PCR	0.918	0.84	0.88
Zhang et al. (37)	2017	China	Asian	85	45	I-IV	40	45	125	miR-222	Serum	qRT-PCR	0.876	0.74	0.90
Kondrotiene et al. (38)	2018	China	Asian	58	45	I-IV	40	45	98	miR-221	Serum	qRT-PCR	0.650	0.77	0.50
Kondrotiene et al. (38)	2018	China	Asian	58	45	I-IV	40	45	98	miR-222	Serum	qRT-PCR	0.821	0.61	0.93
Kondrotiene et al. (39)	2020	Lithuania	Caucasian	49	48	I-IV	23	50	72	miR-221	Plasma	qRT-PCR	0.612	0.55	0.64
Kondrotiene et al. (39)	2020	Lithuania	Caucasian	49	48	I-IV	23	50	72	miR-222	Plasma	qRT-PCR	0.711	0.61	0.78
Kondrotiene et al. (39)	2020	Lithuania	Caucasian	49	48	I-IV	57	45	106	miR-221	Plasma	qRT-PCR	0.792	0.74	0.74
Kondrotiene et al. (39)	2020	Lithuania	Caucasian	49	48	I-IV	57	45	106	miR-222	Plasma	qRT-PCR	0.783	0.67	0.70
Shen et al. (40)	2020	China	Asian	40	46	I-IV	40	46	80	miR-221	Tissue	qRT-PCR	0.660	0.84	0.72
Shen et al. (40)	2020	China	Asian	40	46	I-IV	40	46	80	miR-222	Tissue	qRT-PCR	0.653	0.62	0.90
Shen et al. (40)	2020	China	Asian	47	46	I-IV	47	46	94	miR-221	Tissue	qRT-PCR	0.803	0.81	0.75
Shen et al. (40)	2020	China	Asian	47	46	I-IV	47	46	94	miR-222	Tissue	qRT-PCR	0.802	0.78	0.73

NA, Not Available.

### Pooled Diagnostic Performance

**Figure 2:**
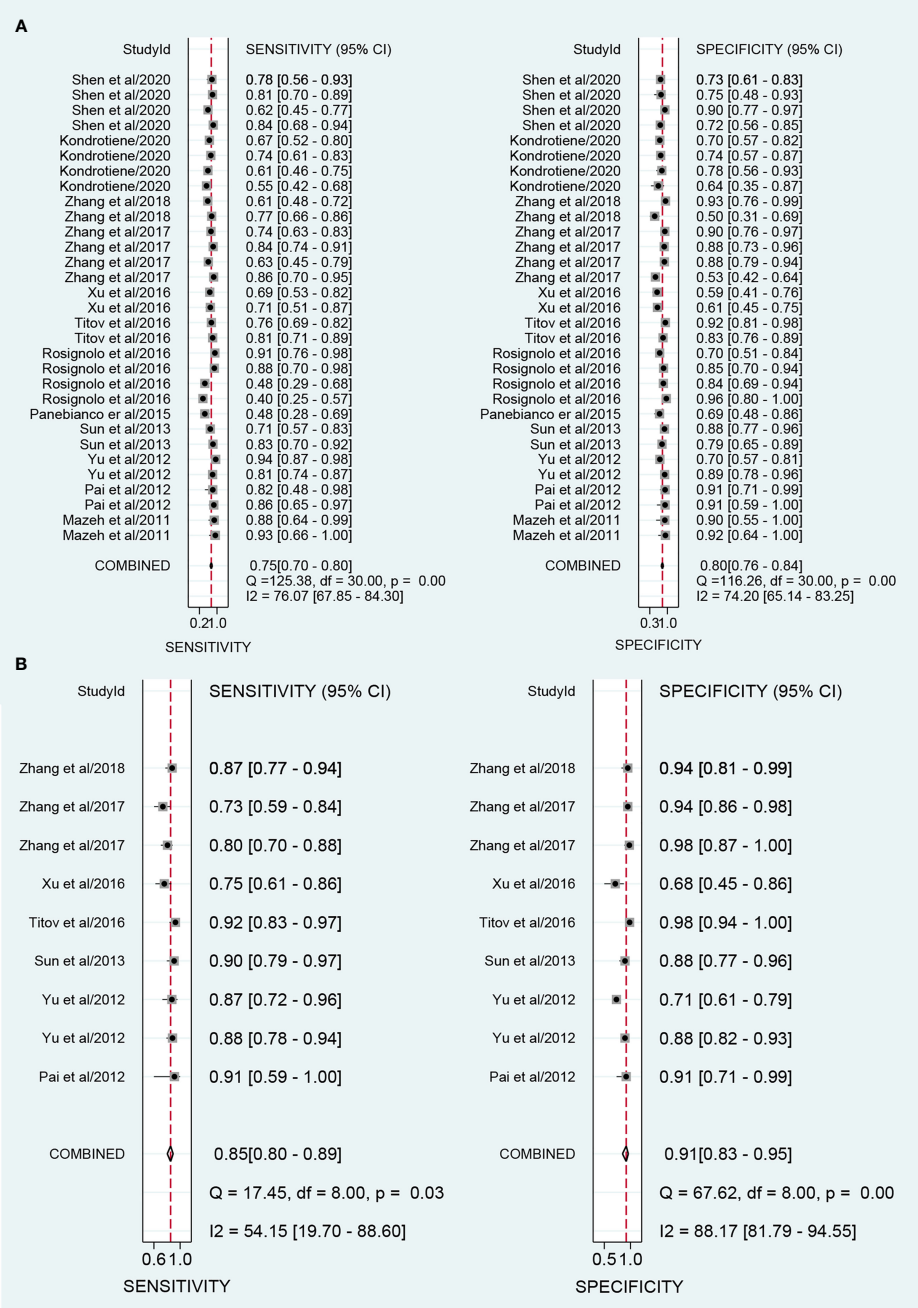
Forest plots of sensitivity and specificity for the distinguishing of papillary thyroid carcinoma (PTC) and controls. **(A)** Forest plots for using one of miR-221/222 in the diagnosis of PTC; **(B)** Forest plots for combination biomarkers containing both miR-221/222 in the diagnosis of PTC.

**Figure 3:**
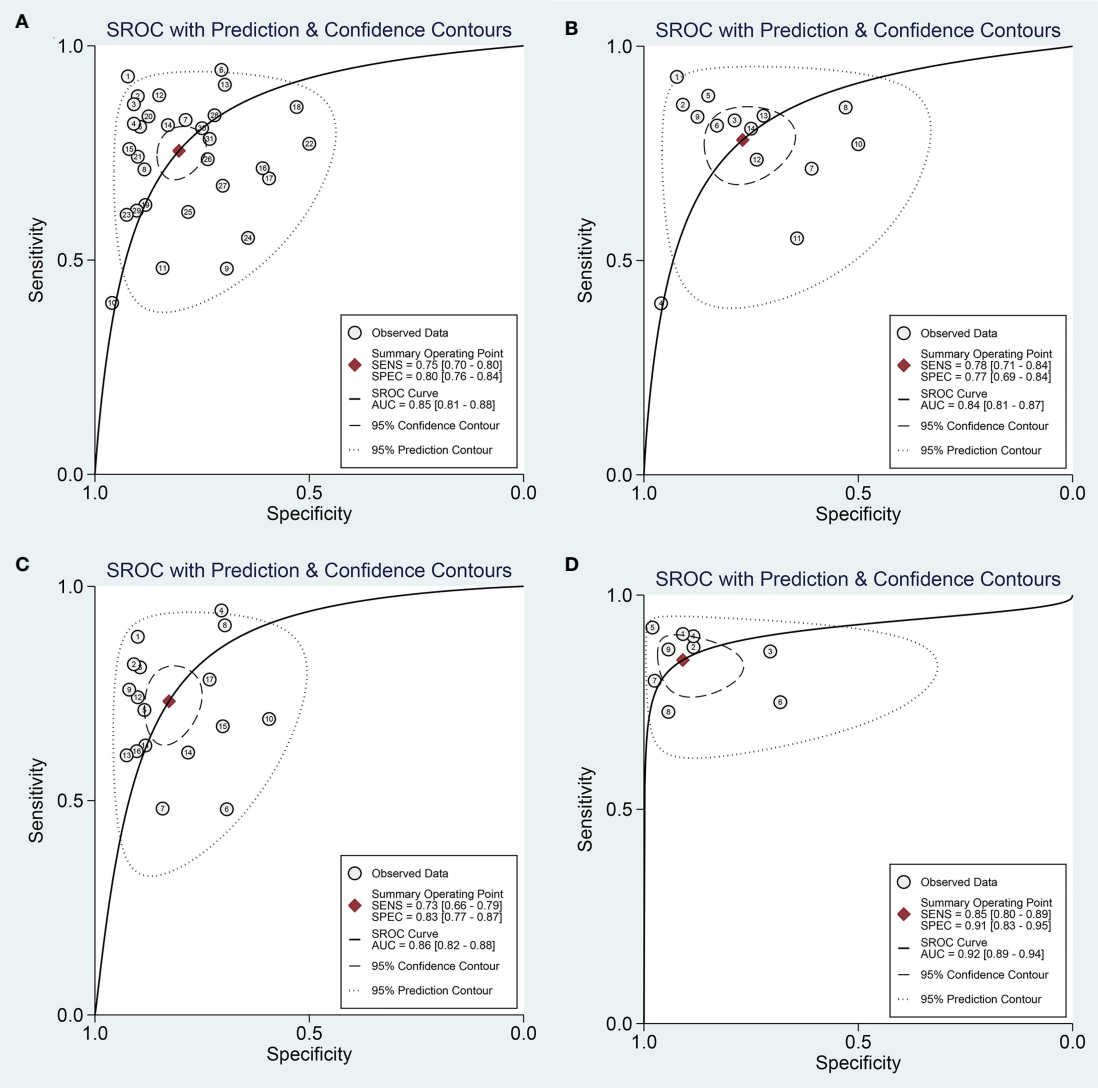
Summary receive operating characteristic (SROC) curve for miR-221/222 in the diagnosis of PTC. **(A)** SROC curve for using one of miR-221/222; **(B)** SROC curve for single miR-221; **(C)** SROC curve for single miR-222; **(D)** SROC curve for combination biomarkers containing both miR-221/222.

The overall sensitivity and specificity of using one of miR-221/222 as diagnostic substrate for PTC in all the studies included were 0.75 (95% CI: 0.70–0.80) and 0.80 (95% CI: 0.76–0.84) respectively with heterogeneity (I^2 =^ 76.07% and 74.20%), when normal tissues or adjacent non-cancerous tissues or nodular goiter were used as the control group. The forest plots for sensitivity and specificity were presented in **Figure 2A**. In addition, from our calculations, the pooled PLR and NLR were 3.9 (95% CI: 3.1–4.8) and 0.30 (95% CI: 0.25–0.37). The DOR was 13 (95% CI: 9–18). The pooled PLR indicated that patients with PTC had a nearly four-fold greater chance of having an elevated miR-221/222 compared with patients without PTC. The pooled NLR meant that the probability of the patient having PTC is 30% if the miR-221/222 is negative. The DOR value meant that someone who was found to be positive for PTC with a high level of miR-221/222 had a 13-fold higher chance of actually suffering from PTC compared with someone with a negative PTC expression. The SROC curve with an AUC of 0.85 (95% CI: 0.81-0.88) is depicted in **Figure 3A**, indicating a relatively high diagnostic performance.

### Subgroup and Meta-Regression Analyses

The threshold effect was one of the major causes of heterogeneity, which occurred due to differences in sensitivity and specificity. Thus, the threshold was firstly analyzed to retrospect the proportion of heterogeneity likely due to threshold effect. As a result, there was no threshold effect illustrating in the present study, which could be revealed by the spearman correlation coefficient value of -0.26 (P= 0.07; P > 0.05).

Then, we searched the following sources for heterogeneity: patient characteristics, geographical location, and sample sources, by conducting subgroup analysis (**Table 2**). In the subgroups for the geographical location, miR-221/222 obtained a better diagnostic accuracy in the Asian populations with the sensitivity of 0.79 (95% CI: 0.74-0.83), specificity of 0.81 (95% CI: 0.74-0.86), and AUC of 0.86 (95% CI: 0.83-0.89), respectively, when compared with the Caucasian populations with the sensitivity of 0.68 (95% CI: 0.58-0.77), specificity of 0.80 (95% CI: 0.74-0.85), and AUC of 0.83 (95% CI: 0.79-0.86). The subgroup analyses were also performed based on different sample sizes. The results suggested that the accuracy of miR-221/222 in large sample size studies were much better than in small sample size studies in distinguishing PTC from controls, with a sensitivity of 0.78 (95% CI: 0.73-0.82) vs. 0.73 (95% CI: 0.64-0.81), specificity of 0.81 (95% CI: 0.74-0.87) vs. 0.79 (95% CI: 0.73-0.84), and AUC of 0.86 (95% CI: 0.83-0.89) vs. 0.83 (95% CI: 0.80-0.86). Furthermore, the studies detecting tissue samples have a little higher diagnostic value than circulating samples with a sensitivity of 0.77 (95% CI: 0.72-0.81) vs. 0.74 (95% CI: 0.66-0.81), specificity of 0.81 (95% CI: 0.75-0.86) vs. 0.80 (95% CI: 0.73-0.86), and AUC of 0.85 (95% CI: 0.81-0.88) vs. 0.84 (95% CI: 0.81-0.87). Pooled studies about miR-221 in PTC exhibited higher diagnostic sensitivity of 0.78 (95% CI: 0.71-0.84) compared with studies concentrating on miR-222, for which the value was 0.73 (95% CI: 0.66-0.79); in contrast, miR-222 was associated with higher specificity of 0.83 (0.77-0.87) compared with the value for miR-221 of 0.77 (0.69-0.84). Overall, miR-222 assays exhibited slightly higher overall diagnostic power than miR-221, for which the AUC values were 0.86 (0.82-0.88) and 0.84 (0.81-0.87), respectively. The SROC curves of miR-221 and miR-222 were plotted at **Figures 3B, C**, respectively.

**Table 2:** Summary table of the diagnostic accuracy of miR-221/222 for PTC.

Analyses	Studies	Sensitivity (95% CI)	Specificity (95% CI)	DOR (95% CI)	PLR (95% CI)	NLR (95% CI)	AUC (95% CI)
miRNA							
miR-221	14	0.78 (0.71-0.84)	0.77 (0.69-0.84)	12 (7-20)	3.4 (2.4-4.7)	0.28 (0.21-0.38)	0.84 (0.81-0.87)
miR-222	17	0.73 (0.66-0.79)	0.83 (0.77-0.87)	13 (8-20)	4.2 (3.2-5.6)	0.32 (0.25-0.42)	0.86 (0.82-0.88)
Sample types							
Blood-based	16	0.74 (0.66-0.81)	0.80 (0.73-0.86)	12 (7-18)	3.7 (2.8-5.1)	0.32 (0.24-0.43)	0.84 (0.81-0.87)
Tissue-based	15	0.77 (0.72-0.81)	0.81 (0.75-0.86)	14 (9-23)	4.0 (2.9-5.5)	0.29 (0.23-0.36)	0.85 (0.81-0.88)
Ethnicity							
Asian	20	0.79 (0.74-0.83)	0.81 (0.74-0.86)	15 (10-23)	4.0 (3.0-5.5)	0.26 (0.22-0.32)	0.86 (0.83-0.89)
Caucasian	11	0.68 (0.58-0.77)	0.80 (0.74-0.85)	9 (5-15)	3.5 (2.6-4.7)	0.39 (0.29-0.54)	0.83 (0.79-0.86)
Sample size							
Large sample size (≥100)	14	0.78 (0.73-0.82)	0.81 (0.74-0.87)	16 (10-24)	4.2 (3.0-5.8)	0.27 (0.22-0.33)	0.86 (0.83-0.89)
Small sample size (<100)	17	0.73 (0.64-0.81)	0.79 (0.73-0.84)	10 (6-18)	3.5 (2.6-4.7)	0.34 (0.25-0.46)	0.83 (0.80-0.86)
Overall	31	0.75 (0.70-0.80)	0.80 (0.76-0.84)	13 (9-18)	3.9 (3.1-4.8)	0.30 (0.25-0.37)	0.85 (0.81-0.88)
Outliers excluded	29	0.75 (0.71-0.79)	0.80 (0.75-0.84)	12 (9-17)	3.8 (3.0-4.8)	0.31 (0.26-0.37)	0.84 (0.80-0.87)
Combination biomarkers	9	0.85 (0.80-0.89)	0.91 (0.83-0.95)	56 (24-132)	9.4 (4.9-18.0)	0.17 (0.12-0.23)	0.92 (0.89-0.94)

DOR, diagnostic odds ratio; PLR, positive likelihood ratio; NLR, negative likelihood ratio; AUC, area under curve; PTC, papillary thyroid carcinomas.

Then, the above potential sources which contributed to heterogeneity were analyzed with a meta-regression. However, meta-regression analysis did not solve the problem of heterogeneity.

### Sensitivity Analyses and Publication Bias

**Figure 4:**
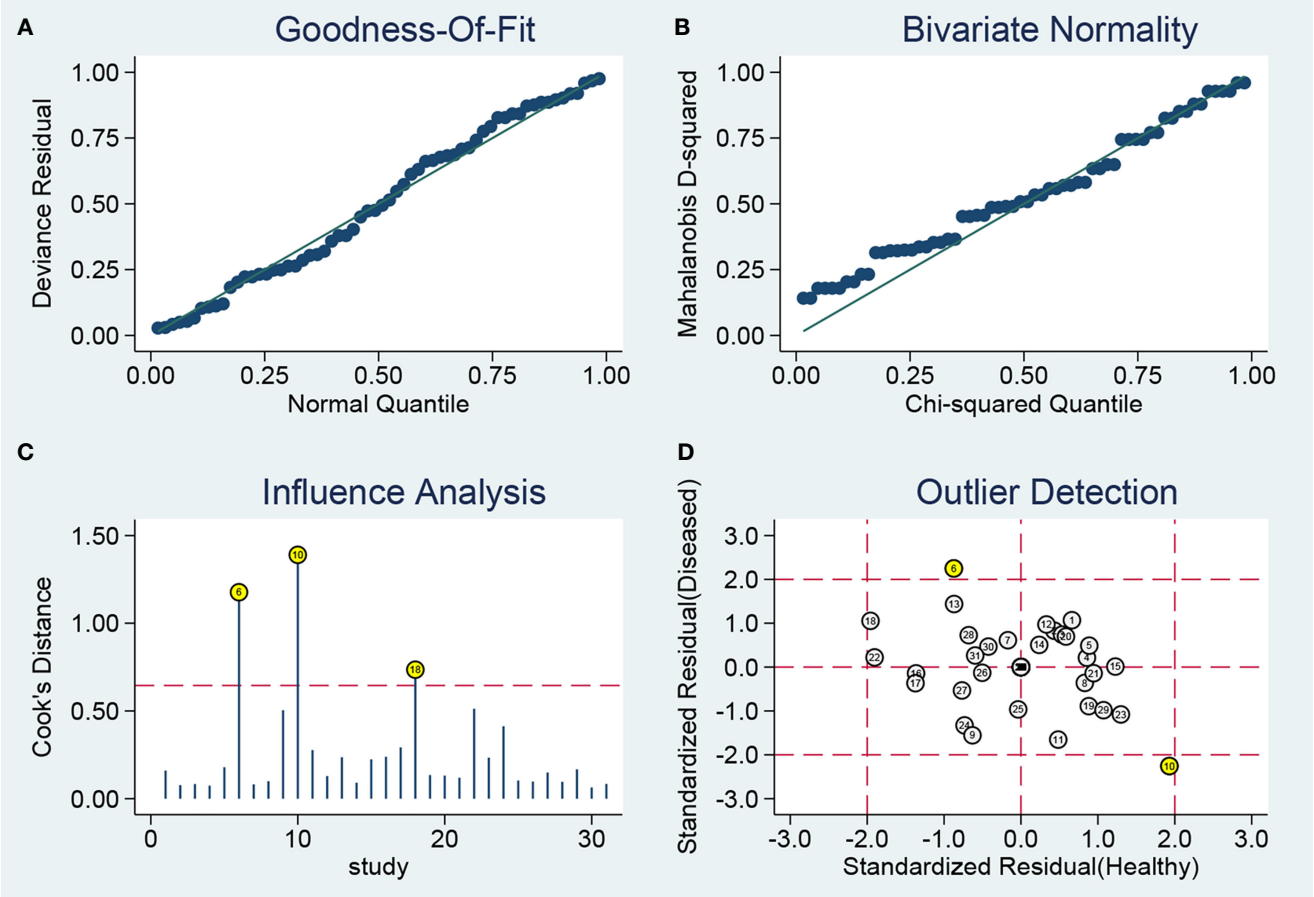
Sensitivity analysis results. **(A)** Goodness-Of-Fit; **(B)** Bivariate Normality; **(C)** Influence Analysis; **(D)** Outlier Detection.

Sensitivity analysis (**Figure 4**) was carried out to explore the influence of single study on overall synthesis result if the data of outliners were removed. After two outliners (31, 34) excluded from the test, the sensitivity and specificity were not changed, PLR decreased from 3.9 to 3.8, NLR increased from 0.30 to 0.31, DOR dropped from 13 to 12, and AUC had minimal change from 0.85 to 0.84. Although the exclusion changed some diagnostic parameters, the combined results remained stable, suggesting that our work was robust.

**Figure 5:**
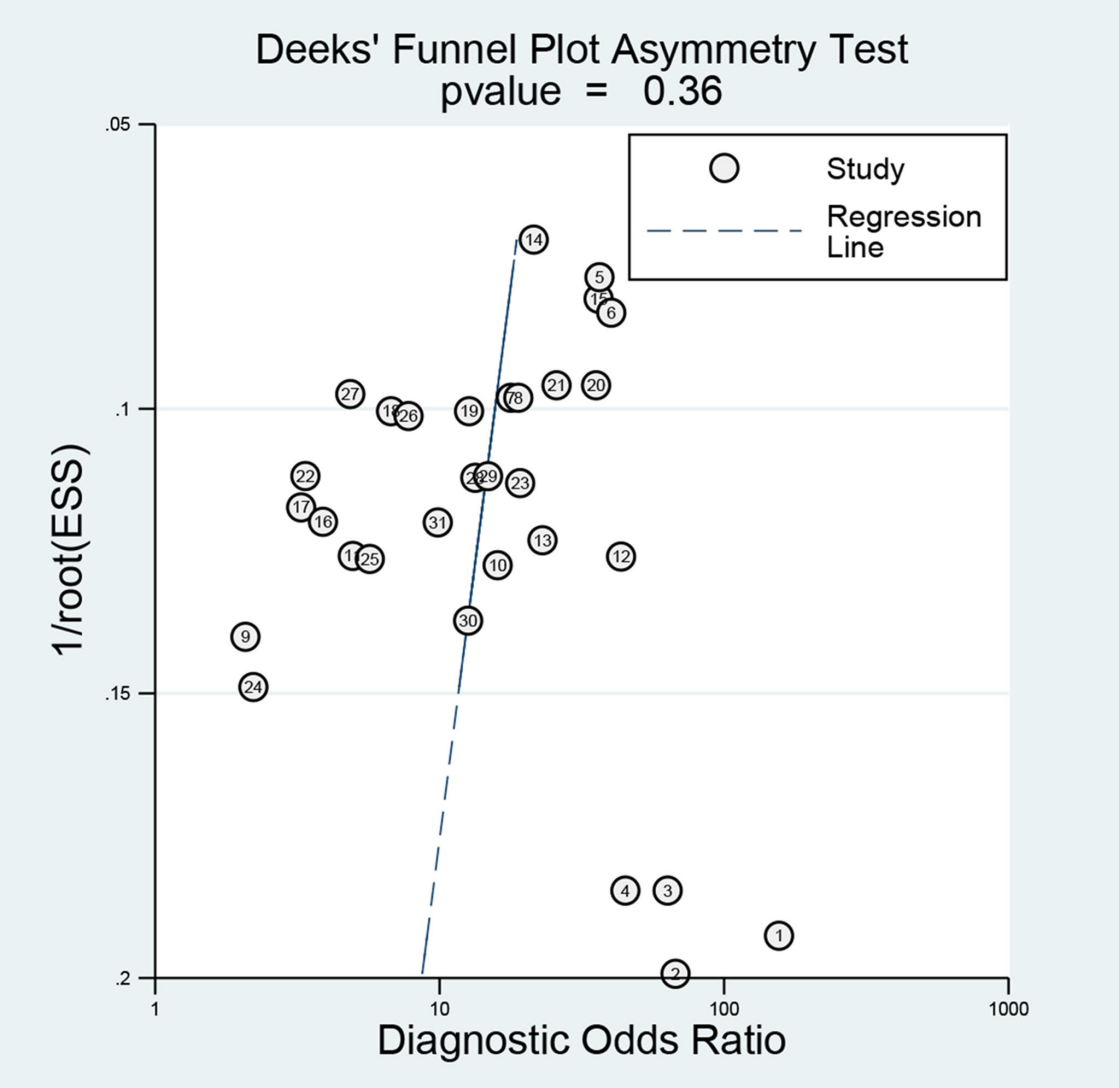
Deek’s plot for the assessment of publication bias.

Furthermore, we evaluated publication bias in the available studies using funnel plots (**Figure 5**). The P-value of 0.36 suggested there is no publication bias. The above tests prove the robustness of this study.

### Combination Biomarkers Containing Both miR-221/222

A total of 9 studies from 7 English language articles illustrated combination biomarkers which contained both miR-221/222 in the diagnosis of PTC (30–32, 35–38) (**Table 3**). The AUC of these combination biomarkers was 0.92 (95% CI: 0.89-0.94) with sensitivity of 0.85 (95% CI: 0.80-0.89) and specificity of 0.91 (95% CI: 0.83-0.95) (**Figure 2B**). The SROC curve was illustrated at **Figure 3D**. Moreover, the pooled PLR, NLR and DOR were 9.4 (95% CI: 4.9-18.0), 0.17 (95% CI: 0.12-0.23), and 56 (95% CI: 24-132), respectively. These findings indicated that combining both miR-221/222 and other miRNAs when used in a diagnostic panel can highly improve diagnostic accuracy of PTC than individual miR-221/222.

**Table 3:** The main features of the included studies on combination markers containing miR-221/222 in the diagnosis of PTC.

Author	Year	Country	Ethnity	Case			Control		Combinations	Source	Methods	AUC	Sensitivity	Specificity
				No.	Age	Stage	No.	Age						
Pai et al. (30)	2012	India	Asian	22	NA	NA	11	NA	miR-221, miR-222	Tissue	qRT-PCR	0.90	0.91	0.91
Yu et al. (31)	2012	China	Asian	106	45	I-IV	95	45	miR-222, let-7e, miR-151	Serum	qRT-PCR	0.92	0.88	0.88
Yu et al. (31)	2012	China	Asian	106	45	I-IV	44	45	miR-222, let-7e, miR-151	Serum	qRT-PCR	0.90	0.87	0.71
Sun et al. (32)	2013	China	Asian	52	45	I-IV	52	45	miR-221, miR-222, miR-146b, miR-181, miR-21	Tissue	qRT-PCR	0.94	0.90	0.88
Titov et al. (35)	2016	Russia	Caucasian	108	53	I-IV	108	53	miR-221, miR-222, miR-21, miR-205, miR-146b, miR-31, miR-187, miR-181b, miR-375	Tissue	qRT-PCR	NA	0.92	0.98
Xu et al. (36)	2016	China	Asian	59	47	NA	15	47	miR-221, miR-222	Tissue	qRT-PCR	0.66	0.75	0.68
Zhang et al. (37)	2017	China	Asian	85	45	I-IV	35	45	miR-221, miR-222, miR-146b	Serum	qRT-PCR	0.90	0.80	0.98
Zhang et al. (37)	2017	China	Asian	85	45	I-IV	40	45	miR-221, miR-222, miR-146b	Serum	qRT-PCR	0.96	0.73	0.94
Zhang et al. (38)	2018	China	Asian	58	45	I-IV	40	45	miR-221, miR-222, miR-146b, miR-21	Serum	qRT-PCR	0.97	0.87	0.94

AUC, area under curve; NA, not available.

### Functional and Pathway Enrichment Analyses of miR-221/222 Targets

**Figure 6:**
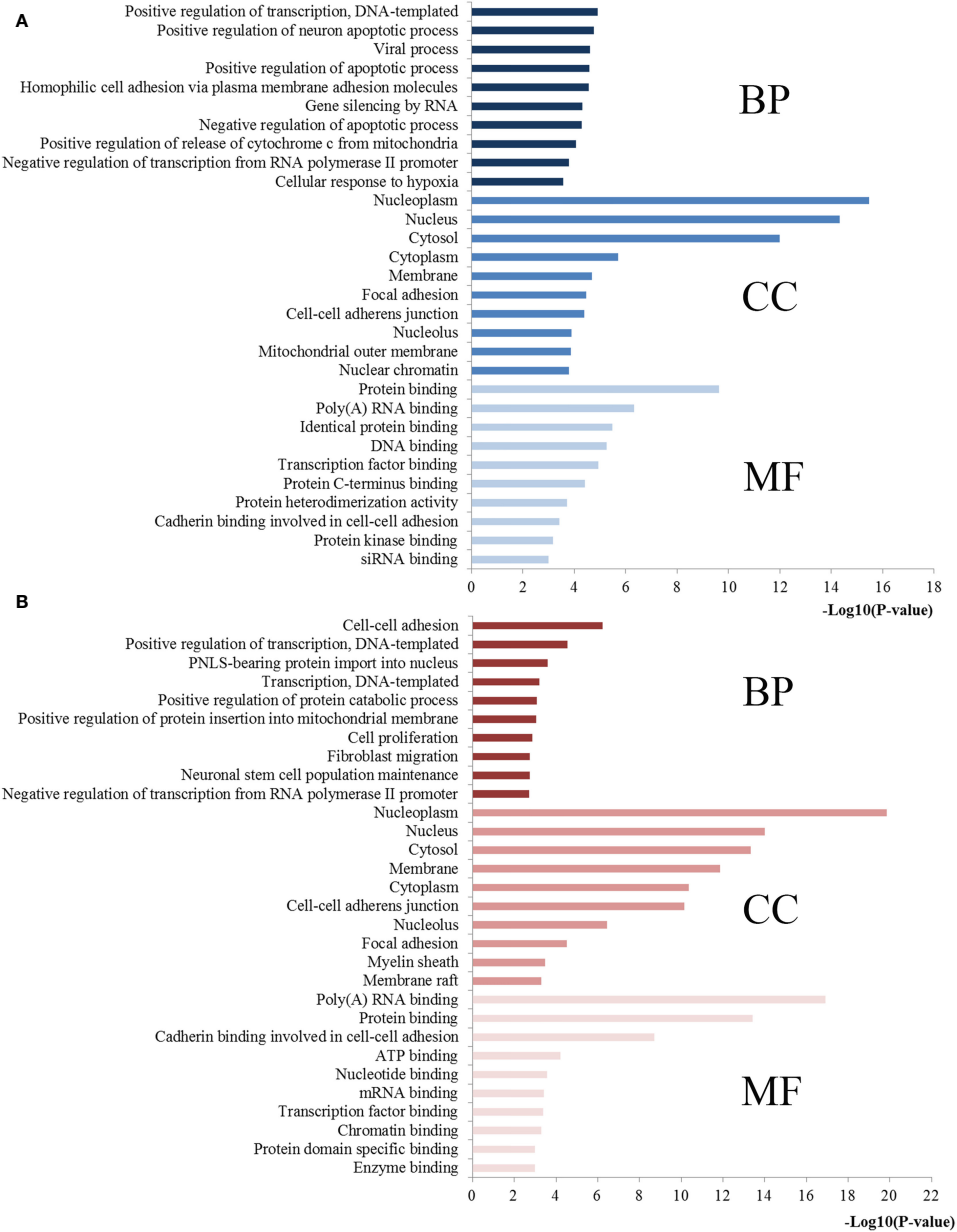
GO annotations of miR-221/222 target genes. **(A)** Top 10 GO items for target genes of miR-221; **(B)** Top 10 GO items for target genes of miR-222. GO, gene ontology; BP, biological processes; CC, cell component; MF, molecular function.

The functional and pathway enrichment analyses were performed to elucidate the function of miR-221/222-targeted genes. The top 10 enriched GO terms of the target genes of miR-221/222 for each analysis were compiled in **Figure 6**. For BP, the target genes of miR-221 were mostly enriched in positive regulation of transcription, positive regulation of neuron apoptotic process, and positive regulation of apoptotic process, while the target genes of miR-222 were highly concentrated in positive regulation of transcription, positive regulation of protein catabolic process, and positive regulation of protein insertion into mitochondrial membrane involved in apoptotic signaling pathway. In terms of CC, the target genes of miR-221 were mainly involved in nucleoplasm, nucleus, cytosol, cytoplasm, and membrane, while the target genes of miR-222 were significantly related to nucleoplasm, nucleus, cytosol, membrane and cytoplasm. Regarding MF, the target genes of miR-221 were obviously associated with protein binding, poly(A) RNA binding, identical protein binding, DNA binding, and transcription factor binding, while the target genes of miR-222 were markedly linked with poly(A) RNA binding, protein binding, cadherin binding involved in cell-cell adhesion, ATP binding, and nucleotide binding.

**Figure 7:**
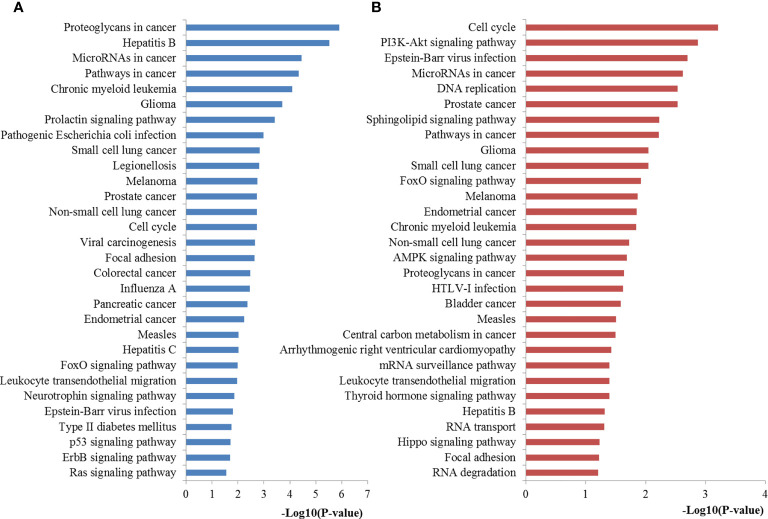
Pathway enrichment results for miR-221/222 target genes. **(A)** Pathways enriched by target genes of miR-221; **(B)** Pathways enriched by target genes of miR-222.

Based on KEGG pathway analysis, the target genes of miR-221 were significantly enhanced for proteoglycans in cancer, miRNAs in cancer, pathways in cancer, cell cycle, FoxO signaling, neurotrophin signaling, Epstein-Barr virus infection, p53 signaling pathway, and Ras signaling pathway. Regarding miR-222, the targets of it were mainly involved in cell cycle, PI3K-Akt signaling pathway, miRNAs in cancer, DNA replication, sphingolipid signaling pathway, pathways in cancer, FoxO signaling pathway, AMPK signaling pathway, proteoglycans in cancer, central carbon metabolism in cancer, mRNA surveillance pathway, thyroid hormone signaling pathway, RNA transport, Hippo signaling pathway, focal adhesion and RNA degradation. The most significantly enriched pathways of miR-221/222 targets analyzed by KEGG analysis were plotted in **Figure 7**.

### PPI Network Analysis and Hub Gene Recognition

To further explore the features and functions of miR-221/222, we conducted biological analyses by constructing the PPI network of their target genes.

**Figure 8:**
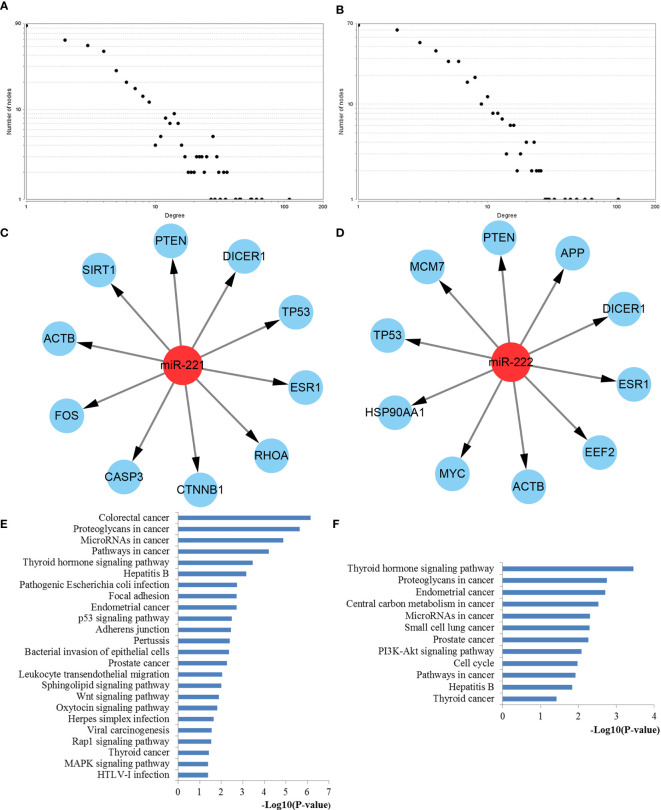
PPI network construction results. **(A)** Degree distributions of nodes for network constructed with miR-221 targets; **(B)** Degree distributions of nodes for network set up with miR-222 targets; **(C)** Top ten hub genes of network for miR-221 targets; **(D)** Top ten hub genes of network for miR-222 targets; **(E)** pathway enrichment results for the identified hub genes of miR-221 targets network; **(F)** pathway enrichment results for the identified hub genes of miR-222 targets network. PPI protein–protein interaction.

The interactions between target genes of miR-221/222, which consisted of 422 nodes and 1613 edges for miR-221, 390 nodes and 1343 edges for miR-222, were downloaded from the STRING database and visualized using Cytoscape to build the PPI networks, respectively. The CytoNCA plugin was used to filter the hub genes associated with PTC by taking the intersections of the results independently identified by three network parameters including betweenness centrality, closeness centrality, and degree centrality. The degree distributions for the network proteins were presented in **Figures 8A, B**. The top ten hub genes for miR-221 included ACTB, CASP3, CTNNB1, DICER1, ESR1, FOS, PTEN, RHOA, SIRT1, and TP53, while the top ten hub genes for miR-222 included ACTB, APP, DICER1, EEF2, ESR1, HSP90AA1, MCM7, MYC, PTEN, and TP53 (**Figures 8C, D**).

### Functional Enrichment and Expression Analysis of Hub Genes

**Figure 9:**
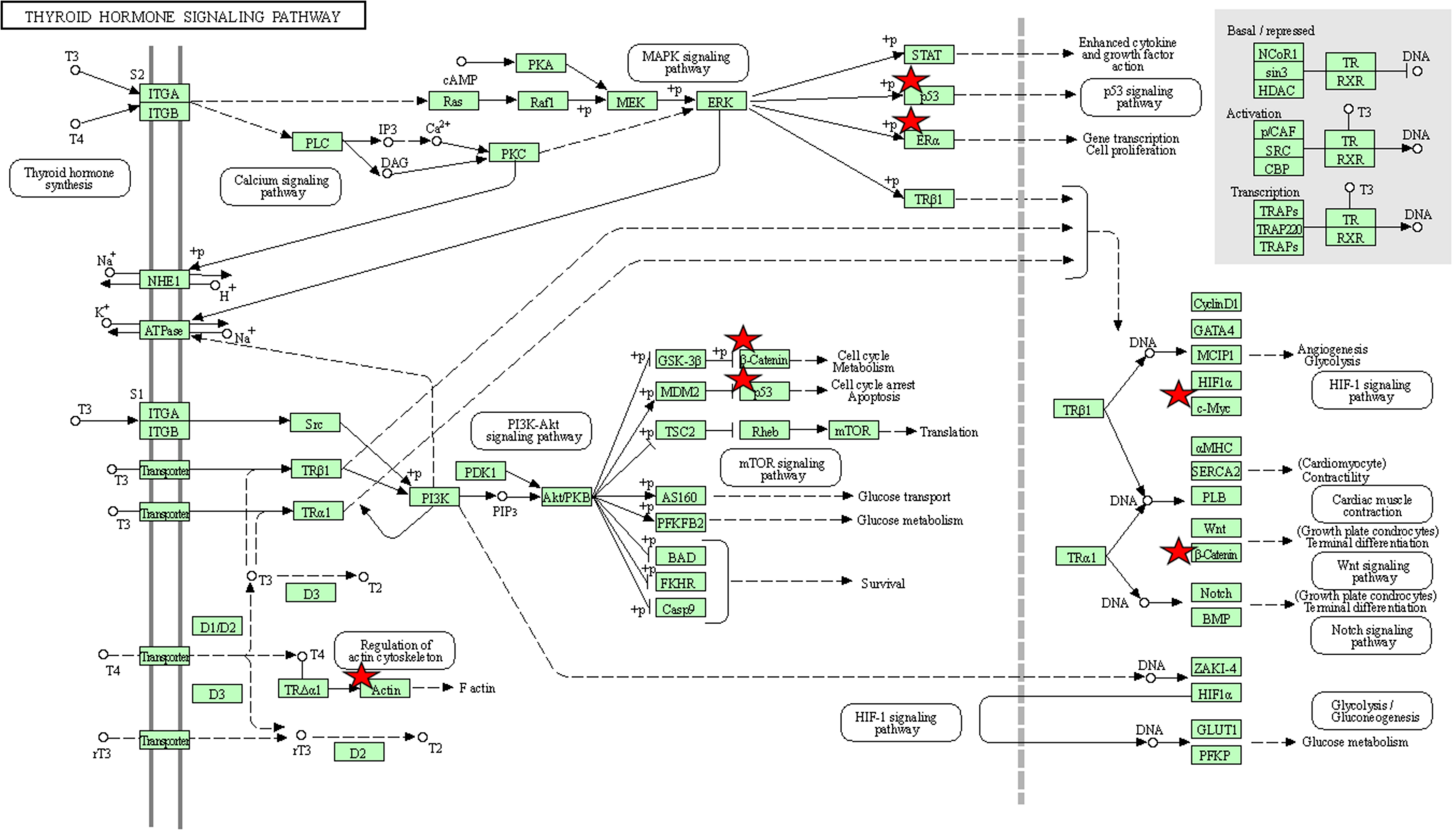
The thyroid hormone signaling pathway enriched in KEGG. Objects with pentagrams are acting locus by mapped genes. KEGG, Kyoto encyclopedia of genes and genomes.

To better understand the roles of hub genes involved in the initiation and progression of PTC, KEGG pathway enrichment analysis was then performed for the hub genes. We found that these hub genes regulated by miR-221 played a significant role in thyroid hormone signaling pathway, proteoglycans in cancer, miRNAs in cancer, pathways in cancer, focal adhesion, p53 signaling pathway, adherens junction, leukocyte transendothelial migration, sphingolipid signaling pathway, wnt signaling pathway, rap1 signaling pathway, thyroid cancer, and MAPK signaling pathway, while these hub genes regulated by miR-222 were mainly involved in thyroid hormone signaling pathway, proteoglycans in cancer, central carbon metabolism in cancer, miRNAs in cancer, PI3K-Akt signaling pathway, cell cycle, pathways in cancer, and thyroid cancer (**Figures 8E, F**). Thyroid hormone signaling may be the most important and direct pathways for miR-221/miR-222 involved in PTC. Thus, we reconstructed this pathway from KEGG as a significant example in **Figure 9**. Interestingly, we found that the enriched thyroid hormone signaling was also closely connected with MAPK, PI3K-Akt, p53, and cell cycle.

**Figure 10:**
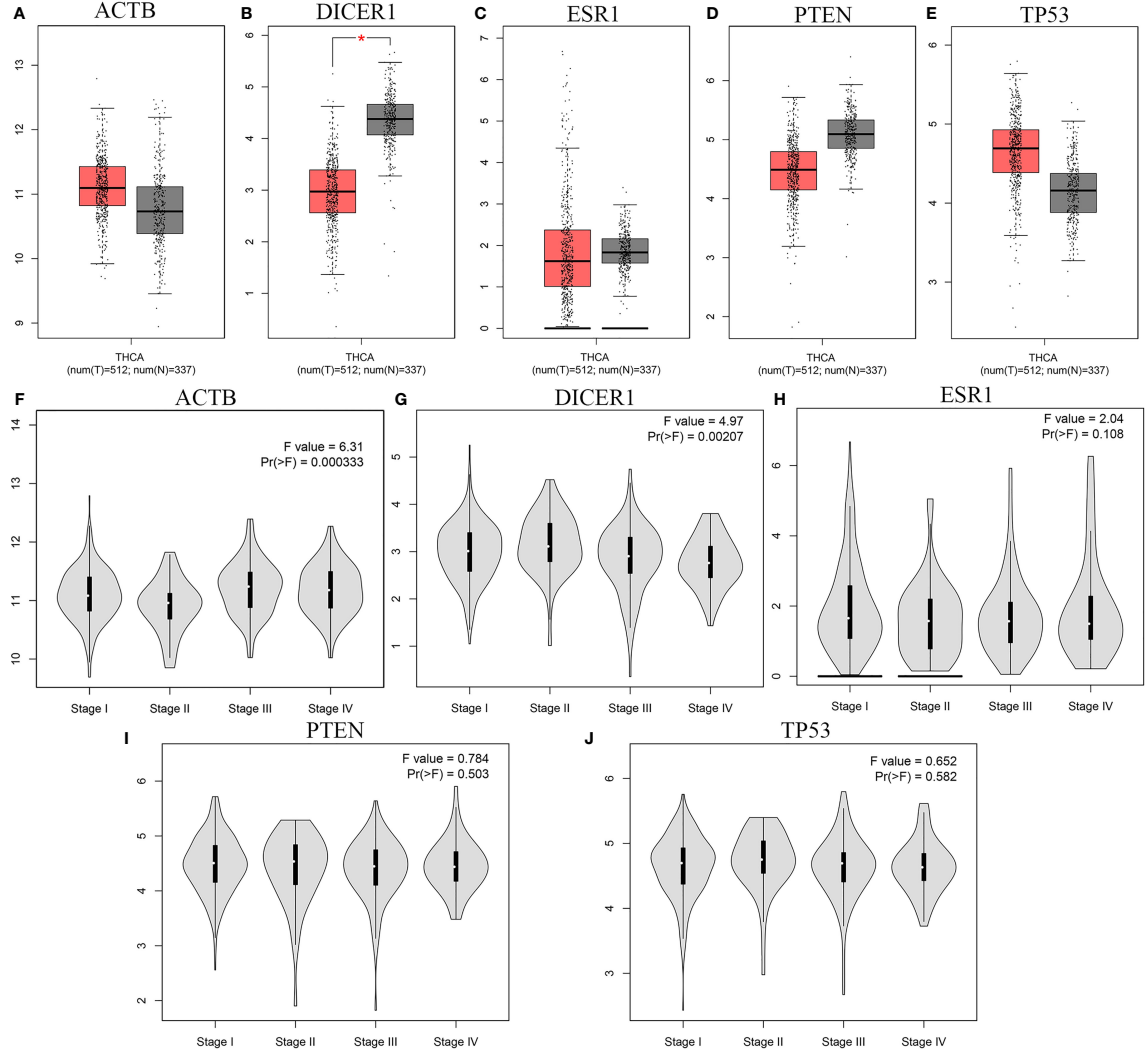
The mRNA expression and the stage-specific expression analysis results of five hub genes. **(A-E)** The mRNA expression of the five hub genes based on TCGA database with GEPIA: **(A)** ACTB, **(B)** DICER1, **(C)** ESR1, **(D)** PTEN, **(E)** TP53; **(F-J)** The stage-specific expressions of hub genes in TCGA *via* GEPIA: **(F)** ACTB, **(G)** DICER1, **(H)** ESR1, **(I)** PTEN, **(J)** TP53. * means statistical significant.

We further validated the 5 overlapped genes between the two lists of top ten hub genes of miR-221/222 based on TCGA database *via* GEPIA: ACTB, DICER1, ESR1, PTEN, and TP53. The results based on GEPIA demonstrated that the mRNA expression level of DICER1 was significantly lower in carcinoma group compared to non-tumor group (P < 0.05). However, there was no significant difference in the mRNA levels of ACTB, ESR1, PTEN, and TP53 between carcinoma group and non-tumor group (P > 0.05) (**Figures 10A–E**).

Meanwhile, we analyzed the correlation between the expression levels of 5 overlapped genes and tumor stage in thyroid cancer patients. The results obtained from GEPIA database demonstrated that the levels of ACTB and DICER1 were associated with tumor stage while the ESR1, PTEN, and TP53 groups did not significantly differ (**Figures 10F–J**).

### Module Analysis and Functional Annotation

**Figure 11:**
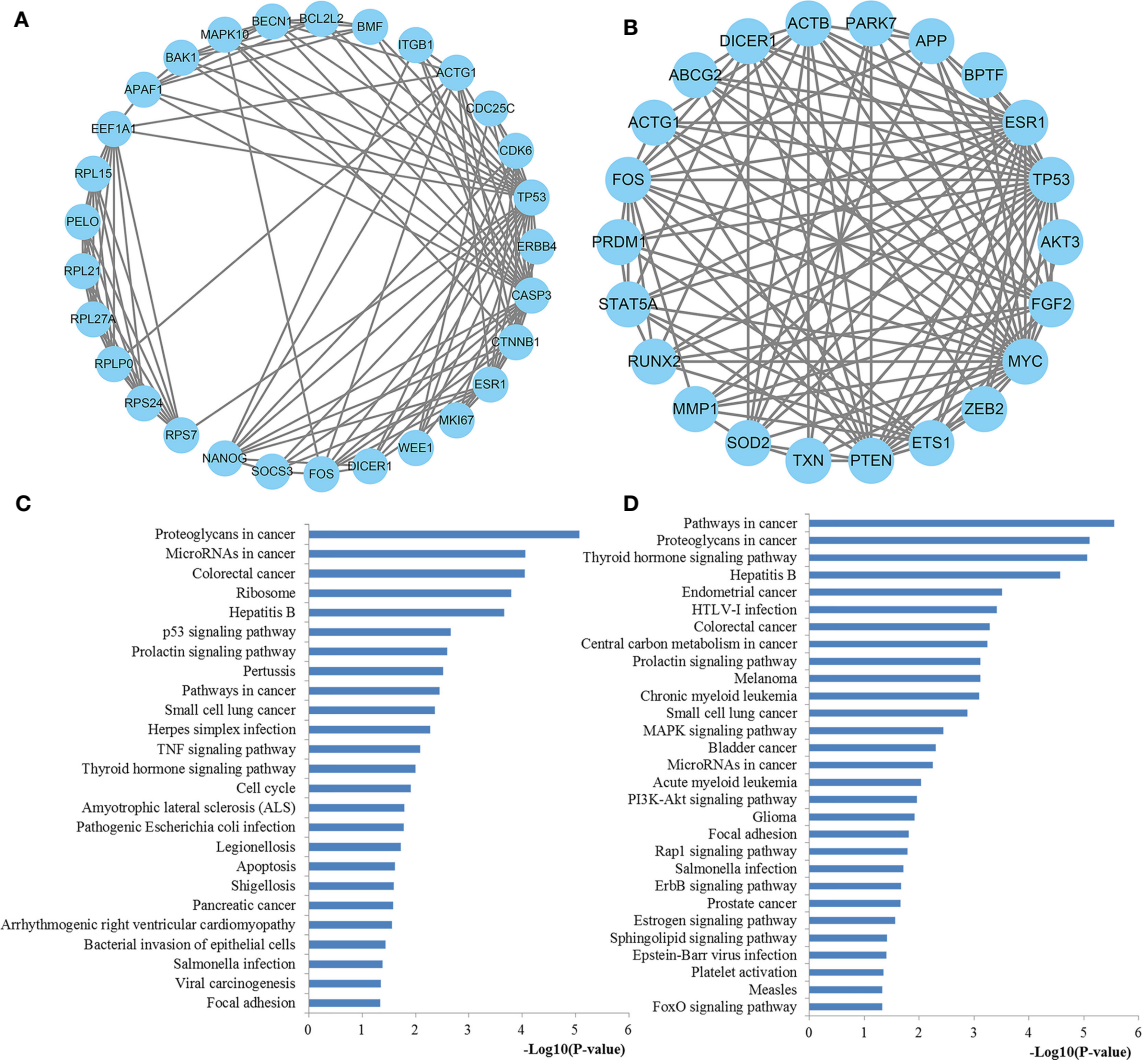
The significant modules from the PPI network. **(A)** The significant module in the PPI network for miR-221 targets; **(B)** The significant module in the PPI network for miR-222 targets; **(C)** Pathways enriched by all the nodes involved in the selected module for miR-221; **(D)** Pathways enriched by all the nodes involved in the selected module for miR-222. PPI protein–protein interaction.

The PPI network was further analyzed by using the MCODE plug-in, and then the most vital gene modules were selected for further analysis. The modules of miR-221 and miR-222 network genes consisted of 29 nodes and 22 nodes, respectively (**Figures 11A, B**). According to the KEGG pathway analysis, the miR-221 network gene module was mainly involved in proteoglycans in cancer, miRNAs in cancer, ribosome, p53 signaling pathway, pathways in cancer, TNF signaling pathway, thyroid hormone signaling pathway, cell cycle, apoptosis, and focal adhesion, whereas the miR-222 network gene module was highly associated with pathways in cancer, proteoglycans in cancer, thyroid hormone signaling pathway, central carbon metabolism in cancer, MAPK signaling pathway, miRNAs in cancer, PI3K-Akt signaling pathway, Rap1 signaling pathway, ErbB signaling pathway, and FoxO signaling pathway (**Figures 11C, D**).

## Discussion

As the incidence and the morbidity of thyroid cancer have increased over the past decades, exploration of effective biomarkers is important for accurately diagnosing thyroid cancer (41). Increasing evidence has repeatedly demonstrated the key involvement of miRNAs in thyroid cancer. Among the miRNAs frequently dysregulated in thyroid cancer, miR-221 and miR-222 are considered of great importance. To date, many studies have reported the possibility of miR-221/222 as non-invasive biomarkers for PTC screening, but diagnostic accuracy values have been inconsistent between these studies. Therefore, we performed this study to investigate the diagnostic efficacy of miR-221/222 in the assessment of PTC by pooling 31 individual studies. Meanwhile, we also explored the potential mechanisms of miR-221 and miR-222 involved in PTC by an integrated bioinformatics study.

The pooled sensitivity and specificity of miR-221/222 for the diagnosis of PTC in the present study was 0.75 and 0.80, respectively. The AUC, which is an indicator of test performance, was 0.85 in our study. Furthermore, the DOR, which is recognized as a combination parameter of sensitivity and specificity, was 13 in the combined analysis. Obvious heterogeneity was observed in the pooled analysis. We selected subgroup analysis, meta-regression analysis and sensitivity analysis to explore the potential sources of heterogeneity. In the subgroup analysis, some interesting results were found. For example, the results of the ethnicity subgroup analysis indicated that the diagnostic accuracy varies among different populations. Therefore, data of different groups of people need to be treated differently. Moreover, although the blood-based miR-221/222 tests might have lower diagnosis accuracy for PTC than tissue-based miR-221/222 tests, its non-invasive and convenient nature may promote its wide application. In particular, diagnostic tests based on large sample size yielded more attractive overall results compared with investigations with small sample size, indicating large-scale and stratified studies are recommended to further confirm these findings. Our data showed that the miR-221 had a higher sensitivity than miR-222 while miR-222 was associated with higher specificity than miR-221. However, there was no significant difference regarding the diagnostic performance between miR-221 and miR-222. Two deviated studies were identified in influence analysis and outlier detection. However, similar results remained in the pooled estimates between the data pooling with and without outliers, indicating that the main outcomes of our study were highly convincing. The pooled results indicated that miR-221 and miR-222 are potential novel and useful biomarkers with suitable sensitivity and specificity that may be utilized for diagnosis of PTC.

Previous clinical studies have reported that miR-221 and miR-222 may be applied as non-invasive biomarkers for PTC. However, they have not pointed that why miR-221 and miR-222 possessed such biomarker characteristics (12). In this study, we have confirmed miR-221 and miR-222 can assist in the diagnosis of PTC. Moreover, we also performed an integrated bioinformatics study to explore the potential mechanism of miR-221 and miR-222 involved in PTC including GO, pathway and PPI analysis. Most GO terms enriched by miR-221 and miR-222 target genes were both significantly associated with the regulation of key biological activities at the BP level, fundamental cell structures at CC level along with the binding functions such as protein binding, poly(A) RNA binding at MF level. Moreover, the target genes of miR-221 and miR-222 were significantly associated with some important pathways which were highly involved in the occurrence and development of thyroid cancer. For example, the miRNAs in cancer, and pathways in cancer signaling pathways demonstrated the direct associations among miR-221/222 and thyroid cancer. Importantly, miR-221/222 may participate in the pathological behavior of thyroid cancer through thyroid hormone signaling, which plays a critical role in not only regulating the physiological activities of normal cells but also stimulating cancer cell proliferation *via* dysregulation of molecular and signaling pathways (42). Proteoglycans in cancer pathway has been a well-studied pathway which plays an irreplaceable role in mediating cell behavior, cell signaling, and cell matrix interactions in both physiological and pathological conditions (43). Cell cycle, recognized as an important pathway, influences almost every aspects of cellular events including cancer cell growth, differentiation, invasion, and metastasis (44). Visone et al. revealed that miR-221 and miR-222 both overexpressed in PTC, and they can regulate p27Kip1 protein levels and cell cycle (45). Moreover, Liu et al. also found that miR-221-3p has a binding site with lncRNA GAS5, which could regulate thyroid carcinoma cell cycle and proliferation by target miR-221-3p/CDKN2B axis (46). It is well established that FOXO signaling is highly involved in extensive physiological processes and various pathological conditions, containing cardiovascular disease, cancer, diabetes and chronic neurological diseases (47). Recent evidence revealed that Epstein-Barr virus (EBV) infection may play a role in the development of thyroid cancer especially in younger ages (48). As one of the most well-studied pathways, p53 signaling pathway has been implicated in multiple aspects of biological processes, including apoptosis, cell cycle arrest, senescence, metabolism, differentiation and angiogenesis (49). Perdas et al. previously summarized the state of knowledge about miRNAs, BRAF and p53 mutation in the development of PTC and revealed that they may play important roles in the pathogenesis of PTC (50). Ras signaling is the most frequently activated pathway across human cancers and represents critical clinical targets for the design and development of pharmaceutically active agents, including anticancer agents (51). Cavedon et al. found that miR-224 is up-regulated in RAS-mutated medullary thyroid cancers (MTC) and in patients with a better survival outcome and could serve as an independent prognostic biomarker in MTC patients (52). Increasing evidences suggest that PI3K/AKT pathway plays an important role in the pathogenesis of various types of cancers including thyroid cancer and this signaling pathway has been identified to be a critical target in cancer treatment (53). In thyroid cancer, extensive studies have demonstrated that miRNAs could inhibit proliferation, invasion, and migration of PTC by targeting key genes *via* the PI3K/AKT pathway (54). DNA replication is a cell signaling pathway that is activated in response to perturbed replication and is essential to couple genome duplication and cell division in establishing and maintaining cellular differentiation programs (55). The mRNA surveillance was the rapid degradation of mRNAs that contain a premature stop codon and assessed the quality of mRNAs to ensure that they are suitable for translation, playing an important part in every aspects of biological processes (56). RNA transport and RNA degradation are two important pathways that are essential for controlling expression levels of all RNAs and thus have a central role in cellular activities including cell growth, proliferation, invasion, and cell metabolism (57, 58). The Hippo signaling pathway is an evolutionarily conserved pathway involved in tissue development and regeneration that controls organ size by regulating cell proliferation and apoptosis (59). Liu et al. indicated that miRNAs could promote anoikis resistance and lung metastasis through inactivating hippo signaling in thyroid cancer (60). Focal adhesion kinase (FAK) has been known as cytoplasmic non-receptor protein tyrosine kinase that plays an important part in cancer cell adhesion, survival, proliferation, and migration through both its enzymatic activities and scaffolding functions (61). These enriched GO terms and pathways may illustrate the potential mechanisms of miR-221/222 involved in PTC or thyroid cancer.

Since miRNAs regulate a wide array of target mRNAs *via* 3’-UTR binding and subsequent translational repression, we performed a PPI network analysis of all the target genes of miR-221/222 to evaluate their associations and to identify key target genes. Based on the PPI network, top ten hub genes of miR-221 and miR-222 were extracted, respectively. KEGG pathway analysis demonstrated that the hub genes have an important function in thyroid cancer initiation and advancement through a series of pathways. Most of these pathways have been confirmed by previous biological experiments according to PubMed citations. Notably, the hub genes regulated by miR-221 and miR-222 were both significantly associated with thyroid hormone signaling pathway and thyroid cancer, strongly demonstrating that miR-221/222 participated in the initiation and progression of thyroid cancer through these two important pathways. In addition to these pathways, the key hub genes of miR-221/222 were also associated with leukocyte transendothelial migration, wnt signaling pathway, rap1 signaling pathway, MAPK signaling pathway, and central carbon metabolism in cancer. Increasing evidence supports a concept that leukocyte transendothelial migration is a vital process in inflammation and the immune response (62). Wnt signaling is one of the key signaling pathways that affects a variety of physiological activities such as growth, differentiation and migration during development and homeostasis (63). Aberrant Wnt signaling has been reported in a variety of malignancies including thyroid cancer (64). Fuziwara et al. has shown that miRNA-mediated regulation of Wnt signaling is altered in PTC (65). Rap1 signaling is important for regulating basic cellular functions, cellular migration, and polarization. Through its interaction with other proteins, Rap1 signaling also have fundamental roles during cell invasion and metastasis in different cancers (66). MAPK signaling pathways organize a great constitution network that plays essential roles in the regulation of several physiological processes, like cell growth, differentiation, and apoptotic cell death. Due to the crucial importance of this signaling pathway, aberrant expression of the MAPK signaling cascades contributes to the pathogenesis of diverse human cancer types including thyroid cancer (67). Liu et al. miRNA could mediate regulation of MAPK oncogenic signaling in PTC (68). Central carbon metabolism in cancer is a vital pathway that play important role in cancer cell metabolism and may affect the carcinogenesis of PTC (69). We then performed module analysis of the PPI network. The results indicated that the significant modules of the networks constructed by miR-221 and miR-222 targets were highly linked with thyroid hormone signaling pathway and other well-studied pathways which were closely related to the initiation and progression of thyroid cancer according to the above discussions. These results demonstrated that miR-221 and miR-222 were highly associated thyroid cancer again.

Although the different locations on chromosomes and different targets lists, the genes regulated by miR-221 and miR-222 were enriched into similar GO terms pathways, modules, or networks and became more consistent at the functional enrichment levels. According to previous practice (70), functionally related genes usually emerge a coordinated expression to play their roles in the same functional pathways and modules, revealing that miR-221 and miR-222 may have a synergistic effect in the occurrence and development of PTC and may be helpful for the diagnosis of PTC. Meanwhile, our previous studies have demonstrated that miRNA combination biomarkers may be more effective in the diagnosis of solid tumors (71–73). Therefore, we also evaluated the diagnostic performance of combination biomarkers containing miR-221/222. The results revealed that the combination of miR-221/222 and other miRNAs is a more robust indicator of PTC than miR-221/222 alone. These findings may provide us with a foundation for the establishment of an easy, non-invasive, and effective model for the diagnosis of PTC.

As we all know, tissue-based and blood-based miRNAs are different. In our study, although the tissue-based miR-221/222 have the highest diagnostic sensitivity specificity, and AUC compared with blood-based miRNAs, blood-based miRNAs also exhibited high diagnostic value and could be applied in a clinical contex based on its non-invasive and convenient nature. Meanwhile, they will play complementary roles in the diagnosis of the PTC. However, the pooled analysis by combining the two sample sources may bring up the issue of study heterogeneity to some extent. Thus, more interpretation attention of the study results should be concentrated on the subgroup analysis results and further large-scale prospective studies are warranted to focus on certain sample sources.

Nevertheless, there were some limitations in our study. First, the cut-off values of miR-221/222 were different in the included studies, which may cause potential heterogeneity. Second, our studies only concentrated on English researches and there were no other languages, As a result, the data collection may be incomplete. Afterwards, most of the populations in our studies were Asians and Caucasians, which might have a bias towards this population. Finally, although most of the results from bioinformatics analysis have been verified by recent literature report in PubMed citations, they still need further confirmation by biological experiments.

Despite these limitations, our study has several important strengths compared with the previous studies. We summarized the diagnostic and clinical role of miR-221/222 in PTC and demonstrated that they could be potential non-invasive biomarkers for PTC. Moreover, we found that combining miR-221/222 and other miRNAs can improve diagnostic accuracy than individual miR-221/222. Besides, we also evaluated the potential mechanisms of miR-221/222 in thyroid cancer by an integrated bioinformatics analysis, which is a novelty of the study, since no previous diagnostic study has been done to investigate the question why miR-221/222 could serve as diagnostic biomarkers in patients with PTC. Some interesting findings from our study may help guide the future research direction. In particular, we found that miR-221/222 may specifically exert important functions in PTC through thyroid hormone signaling pathway and thyroid cancer signaling pathway. Although other pathways did not have the direct associations with PTC, they were highly involved in cancer initiation and progression and maybe also associated with PTC, which should be validated by biological experiments. The bioinformatics could help us understand the overall biomarker function to some extent.

## Conclusion

In conclusion, as non-invasive biomarkers, miR-221 and miR-222 have a promising future for the diagnosis of PTC. Combination biomarkers containing both miR-221/222 may further improve the diagnostic accuracy than individual miR-221/222. Moreover, miR-221/222 may play a significant role in the initiation and progression of PTC through thyroid hormone signaling pathway and some other key pathways by regulating some key genes. However, further large-scale and stratified studies are required to confirm our observations and promote them into clinical application.

## Data Availability Statement

The raw data supporting the conclusions of this article will be made available by the authors, without undue reservation.

## Author Contributions

SC, JM, and YW carried out the studies, participated in collecting data, and drafted the manuscript. YC and LX performed the statistical analysis and participated in its design. XC and YY helped to draft the manuscript. QP conceived of the study, and took part in its design and coordination. All authors read and approved the final manuscript.

## Funding

This work was supported by the National Natural Science Foundation of China (81902715), the Natural Science Foundation of Jiangsu Province (BK20180195), Training project of “national tutor system” for young health talents in Suzhou (SC), and Medical talent of Suzhou (SC).

## Conflict of Interest

The authors declare that the research was conducted in the absence of any commercial or financial relationships that could be construed as a potential conflict of interest.

## Publisher’s Note

All claims expressed in this article are solely those of the authors and do not necessarily represent those of their affiliated organizations, or those of the publisher, the editors and the reviewers. Any product that may be evaluated in this article, or claim that may be made by its manufacturer, is not guaranteed or endorsed by the publisher.
